# Identifying Communities at Risk for Poor Health using Multidimensional vs. Unidimensional Neighborhood Disadvantage Indices

**DOI:** 10.64898/2026.08.06.26359856

**Published:** 2026-08-10

**Authors:** Philippa Clarke, Kimberly Rollings, Robert Melendez, Kate Duchowny, Lindsay Gypin, Grace Noppert

**Affiliations:** 1 Institute for Social Research, University of Michigan, Ann Arbor, MI, USA; 2 Department of Epidemiology, School of Public Health, University of Michigan, Ann Arbor, MI, USA

**Keywords:** Neighborhood, socioeconomic status, disadvantage, deprivation, United States

## Abstract

**Background:**

Neighborhood disadvantage indices used in public health research and policy include multiple economic, social, and housing items. However, research has failed to question whether it is necessary to include a multitude of economic, social, and housing variables in a single index. The purpose of this work was to examine three different neighborhood indices: a multidimensional disadvantage index, a unidimensional disadvantage index, and a unidimensional affluence index, and examine their performance with respect to distinguishing between healthy and unhealthy census tract neighborhoods in the United States.

**Methods:**

The 2022 disadvantage and affluence indices came from the National Neighborhood Data Archive, which are derived from census tract data from the American Community Survey 5-year estimates (2018-2022). The multidimensional disadvantage index included seven economic, social (e.g., single parent households), and housing items; the unidimensional disadvantage index included three poverty and income items; the unidimensional affluence index included 3 items capturing greater social and economic resources. Data on neighborhood health status (census tract prevalence of obesity, diabetes, and coronary heart disease) was obtained from the Population Level Analysis and Community EStimates database for 2022 and linked to the disadvantage and affluence indices for 83,522 census tracts. Contingency tables examined the degree of correspondence in quintiles across the three different indices and the corresponding disease prevalence in each cell. Generalized linear mixed models regressed the disease prevalence variables on index quintiles to determine the predicted prevalence of disease across the disadvantage gradient for each index.

**Results:**

Compared to the unidimensional disadvantage and affluence indices, the multidimensional disadvantage index underestimated disease burden in the most disadvantaged census tracts, and overestimated disease burden in the least disadvantaged tracts.

**Conclusions:**

Using a disadvantage or affluence index with a more parsimonious set of items would have greater precision in identifying communities at risk for poor health.

## INTRODUCTION

Research has shown that residence in socioeconomically disadvantaged neighborhoods is associated with poor health.(1-4) Neighborhood disadvantage (also termed area-level deprivation or vulnerability) is hypothesized to shape health directly (e.g., through stress processes) (5,6) and indirectly (e.g., through a lack of resources to promote healthy behaviors, few opportunities for employment and education, and poor quality housing) (7-11). Area-level disadvantage is typically measured using administrative data on social, economic, and housing characteristics at local geographies (e.g., United States (US) census tract counts of people in poverty). While some research has relied on single indicator variables to capture disadvantage, composite measures that combine multiple social, economic, housing, and demographic variables in a single index have been shown to be more strongly associated with health outcomes (12,13). Composite indices capture the overlapping contexts that characterize local neighborhoods. Using a composite index in statistical analysis also avoids the risk of multicollinearity when using several related individual variables in the same model.

There are numerous composite disadvantage indices used in US clinical and public health research (13-15). These indices include multiple economic, social, and housing variables (ranging from 7-29 variables) at the local area level (e.g., census tract, block group), which are combined to create a single multidimensional index of disadvantage (13,16). For example, the Area Deprivation Index (ADI), one of the most widely used indices in clinical and health policy work, includes 17 census block group indicators capturing economic characteristics (family income, poverty), social characteristics (education, unemployment, single parent families) and housing characteristics (home ownership, home value, mortgage and rent, household crowding, plumbing, etc.) (17,18). Other commonly used indices, such as the Childhood Opportunity Index (COI)(19), the Social Vulnerability Index (SVI) (20), and the Social Deprivation Index (SDI) (21), capture similar economic, social, and housing items in their index construction in addition to other sociodemographic and environmental indicators (e.g., age structure, transportation, industrial pollutants). However, little research has questioned the necessity of including a multitude of economic, social, and housing variables in a neighborhood disadvantage index, or whether a more parsimonious index is sufficient to understand health outcomes.

A growing number of studies have systematically compared disadvantage indices to evaluate their ability to discriminate between good and poor health at the local area level (22-27). For example, Lou et al (2023) compared five US disadvantage indices on their relationships to 24 diverse health outcomes.(28) They found that area-level poverty, education, and employment were largely driving neighborhood effects on physical health, mental health, and mortality outcomes. Variables capturing neighborhood housing quality, demographic composition (language, minority status) and transportation (vehicle ownership) were less strongly associated with health, suggesting that including multiple social and environmental variables in disadvantage indices may be unnecessary, and could diminish their ability to identify local areas at risk for poor health.

Current health research on neighborhood disadvantage has also tended to overlook the concept of neighborhood affluence. Distinguished from non-disadvantaged neighborhoods by their large share of highly educated adults in professional occupations, affluent neighborhoods have the social and economic resources to promote and sustain local institutions that benefit the health of the neighborhood as a whole (29,30). This upper end of the neighborhood socioeconomic continuum, neighborhood affluence, has often been shown to be more important for health outcomes than neighborhood disadvantage (30-33). Yet, many disadvantage indices include indicators of affluence in their construction (e.g., ADI includes a variable for white collar occupations; COI includes variables for college education and high-skill employment), preventing the ability to distinguish the separate effects of disadvantage and affluence on health outcomes.

The purpose of this work was to examine three different neighborhood indices: a multidimensional disadvantage index (including economic, social, and housing variables), a unidimensional disadvantage index (including only economic variables), and a unidimensional affluence index, and examine their performance with respect to distinguishing between healthy and unhealthy census tract neighborhoods in the US. We evaluated the association between each of the three indices and the neighborhood prevalence of three costly and preventable adult health conditions (obesity, diabetes, and coronary heart disease) that have repeatedly been shown to be patterned by area-level disadvantage (8,34). We hypothesized that a multidimensional index would perform less well in identifying communities at risk for poor health compared to a unidimensional disadvantage or affluence index.

## METHODS

This is a national, cross-sectional study of US census tracts in 2022.

### Data and Measures

#### Neighborhood Disadvantage and Affluence

We used three indices from the National Neighborhood Data Archive (NaNDA) at the census tract level (35). Census tracts have a population size between 1,200 and 8,000 people, with an average size of 4,000 people, and are the preferred geographic level for studying the health effects of neighborhood disadvantage (14). Census tracts are also less likely to have missing data on income-related items compared to smaller geographic areas (i.e., block group), which are frequently suppressed by the Census Bureau, requiring imputation for indices created at the block group level. We used NaNDA’s 2022 disadvantage and affluence indices, which are derived from the US Census Bureau’s American Community Survey (ACS) 5-year estimates (2018-2022). These indices include census tract variables of economic, social, and housing characteristics that are combined into a mean index based on a principal components analysis and factor analysis conducted for each Census/ACS data release (36).

The multidimensional disadvantage index is the mean of seven variables: census tract proportions of people with annual income below the federal poverty level; proportion of households receiving public assistance income or food stamps; proportion of families with annual income <$40,000 (the lowest quartile of the national distribution of census tract family income in ACS 2018-2022); census tract proportion of people (age 16+) in the civilian labor force who are unemployed; proportion of people (age 25+) with less than high school degree; proportion of families with children that are single parents; and proportion of housing units that are not owner occupied. The unidimensional disadvantage index is the mean of three variables: census tract proportions of people with annual income below the federal poverty level; proportion of households receiving public assistance income or food stamps; and proportion of families with annual income <$40,000. The unidimensional affluence index is the mean of three variables: census tract proportions of people (age 25+) with a Bachelor’s Degree or higher; proportion of people (age 16+) in the civilian labor force who are employed in professional or managerial occupations (management, business, science, and arts occupations); and proportion of families with annual income >$125,000 (the highest quartile of the national distribution of census tract family income in ACS 2018-2022).

#### Neighborhood Health Status

Neighborhood health status was obtained from the CDC PLACES database for the year 2022 (37). The Population Level Analysis and Community EStimates (PLACES) database (38) includes crude prevalence of common health conditions for adults (age ≥18 years) nationwide at the census tract level since 2015. PLACES provides model-based health estimates using data from the Behavioral Risk Factor Surveillance System (BRFSS), Census decennial population counts and annual county population estimates, and the ACS 5-year estimates. Small-area estimation methods are used to obtain data on 12 health conditions and other health-related measures in US census tracts (37). The 2022 PLACES release includes data for 83,522 census tracts (at 2020 geography) in the 50 states and District of Columbia that have a Census 2020 adult population count ≥50. We used data on three adult health conditions: obesity ((BMI) ≥30.0 kg/m^2^), diabetes, and coronary heart disease (including angina). We used the census tract crude prevalence of these conditions (percent), rescaled to between 0 and 1 for analysis. CDC PLACES data were linked to NaNDA’s disadvantage and affluence indices by census tract Federal Information Processing Standards (FIPS) codes for all 83,522 census tracts at 2020 Census geography.

### Statistical Analyses

Neighborhood indices were categorized into quintiles for analysis. For comparability with the disadvantage indices, affluence was reversed so that the highest quintile represents the least affluent census tracts while the lowest quintile represents the most affluent census tracts. Descriptive statistics (means, standard deviation (SD)), were first used to summarize the prevalence of the three neighborhood health conditions across gradients (quintiles) of each of the three indices. Contingency tables examined the degree of correspondence in quintiles across the three different indices and the corresponding disease prevalence in each cell. Generalized linear mixed models with a logit link and a beta distribution were then used to regress the disease prevalence variables (modeled as proportions) on quintiles of each neighborhood index. A random effect for US state was included to account for unmeasured factors at the state level that could shape the prevalence of disease at the neighborhood level. Models adjusted for population density (persons per square mile) to account for census tract population and urbanicity. All analyses were conducted in SAS (Version 9.4) and RStudio (Version 4.4.2).

## RESULTS

### Univariate and Bivariate Results

Table 1 presents the mean prevalence of the three health conditions in US neighborhoods, overall and by quintiles of the three indices. Overall, the census tract prevalence of obesity, diabetes, and coronary heart disease was 34.4%, 12.4% and 7.0%, respectively (Table 1). There were clear gradients in the prevalence of each health condition across quintiles of the neighborhood indices, with the highest prevalence of disease in the most disadvantaged tracts (Q5) and the lowest prevalence in the least disadvantaged tracts (Q1). However, there were differences in disease prevalence between the highest (Q5) and lowest (Q1) quintiles depending on the disadvantage index used (Table 1). For example, the difference in the prevalence of coronary heart disease in neighborhoods at the highest and lowest quintiles of the unidimensional disadvantage index was 2.2% (8.1 – 5.9%), and 2.9% between neighborhoods in the highest and lowest quintiles of the reverse-affluence index (8.3 – 5.4%). However, there was only a 1% difference in neighborhood coronary heart disease prevalence between tracts at the highest and lowest quintiles of the multidimensional disadvantage index (7.5 – 6.5%). Similarly, the difference in the prevalence of diabetes between neighborhoods at the highest and lowest quintiles of the reverse-affluence index and the unidimensional disadvantage index was 7.4% and 6.9%, respectively, but only 5.5% between the highest and lowest quintiles of the multidimensional disadvantage index.

In order to further quantify these differences, we examined the degree of correspondence in neighborhood quintiles across the three different indices. Table 2 presents contingency tables showing the number of census tracts where quintiles of the disadvantage and affluence indices agreed and where they disagreed in their classification. Supplementary Tables 1–3 present the corresponding disease prevalence (coronary heart disease, diabetes, and obesity, respectively) for each of these cross-classified quintiles. Consider, for example, the 16,907 census tracts classified as the most disadvantaged (Q5) by the multidimensional disadvantage index (Table 2a). The unidimensional disadvantage index also classified 82% (N=13,961) of these tracts as the most disadvantaged (Q5), and the average prevalence of coronary heart disease in these tracts was 7.9% (Supplemental Table 1a). However, in the remaining 18% of tracts that were considered less disadvantaged (Q1-Q4) by the unidimensional index but still the most disadvantaged (Q5) by the multidimensional index, the average prevalence of coronary heart disease was lower (5.7% on average, ranging from 3.9% to 5.8%, Supplemental Table 1a). Similarly, of the 16,907 census tracts classified as the most disadvantaged (Q5) by the multidimensional disadvantage index, only 61% (N=10,272) were classified as the most disadvantaged (Q5) by the reverse affluence index (Table 2b). In tracts where both the reverse affluence and multidimensional disadvantage indices agreed (both Q5), the average prevalence of coronary heart disease was 8.2% (Supplemental Table 1b). But in the remaining 6,635 tracts considered less disadvantaged (Q1-Q4) by the affluence index but highly disadvantaged (Q5) by the multidimensional index, the average prevalence of coronary heart disease was lower at 6.5% (ranging from 4.1% to 7.2%, Supplemental Table 1b). Thus, the multidimensional disadvantage index classified about 40% of US census tracts as highly disadvantaged when in fact they were less disadvantaged in terms of their health status.

At the other end of the disadvantage spectrum, of the 16,907 census tracts classified as the least disadvantaged (lowest quintile Q1) according to the multidimensional disadvantage index, just over half of these tracts (N=8,964) were also classified as the least disadvantaged (Q1) by the reverse affluence index (Table 2b). The average prevalence of coronary heart disease in these tracts was 5.9% (Supplemental Table 1b). But in the remaining 7,943 census tracts classified as the least disadvantaged (Q1) by the multidimensional index (but more disadvantaged [Q2-Q5] by the affluence index), coronary heart disease prevalence was higher (ranging from 6.7% to 8.1%, Supplemental Table 1b). There was greater agreement in the classification of the least disadvantaged tracts (Q1) between the multidimensional disadvantage and unidimensional disadvantage indices (74% of the tracts classified as the least disadvantaged by the multidimensional index were also classified as the least disadvantaged by the unidimensional index, Table 2a), where the mean prevalence of coronary heart disease was 6.1% (Supplemental Table 1a). However, in the remaining 4,400 census tracts classified as the least disadvantaged by the multidimensional index but more disadvantaged (Q2-Q3) by the unidimensional index, the prevalence of disease was much higher (ranging from 7.4% to 9.1%, Supplemental Table 1a), suggesting that these neighborhoods are in fact more disadvantaged in terms of health than the multidimensional index would indicate.

### Regression Results

Results from the generalized linear mixed models regressing each health outcome on each of the three disadvantage indices are presented in Supplemental Table 4. For ease of interpretation, Figures 1-3 present the predicted prevalence from these models (population average census tract prevalence with 95% CI) for obesity, diabetes, and coronary heart disease (respectively) at average population density across quintiles of each of the disadvantage indices (again, affluence is reverse-scored). Overall, across all three indices, the prevalence of obesity, diabetes, and coronary heart disease was highest at greater levels of disadvantage and lowest at lower levels of disadvantage. However, there were differences in the predicted prevalence of disease at each disadvantage quintile depending on the index used. For example (Figure 1), in the least disadvantaged tracts as measured by the multidimensional disadvantage index, there was 2.3% higher prevalence of obesity than in the least disadvantaged tracts as measured by the reverse affluence index (quintile 1 multidimensional disadvantage index = 29.7% (95% CI: 28.7, 30.6) vs. quintile 1 reverse affluence index = 27.4% (95% CI=26.7, 28.2)). Similarly, the prevalence of diabetes was 10% (95% CI=9.5, 10.4) in the least disadvantaged tracts when using the multidimensional disadvantage index, but only 8.8% (95% CI=8.5, 9.2) in the least disadvantaged tracts when using the affluence index (reversed) (Figure 2). For coronary heart disease (Figure 3), the neighborhood prevalence was 6.4% (95% CI=6.2, 6.6) in the least disadvantaged tracts as measured by the multidimensional disadvantage index compared to 5.9% (95% CI=5.7, 6.1) when using the unidimensional disadvantage index, and 5.6% (95% CI=5.5, 5.8) in the least disadvantaged tracts when using the affluence index (reversed). In general, the prevalence of disease in the least disadvantaged tracts tended to be lowest when using the affluence index and highest when using the multidimensional disadvantage index.

At higher levels of neighborhood disadvantage, there was a convergence in the predicted prevalence of disease around the middle quintiles for all three indices. But by the third quintile of disadvantage, the predicted prevalence of disease was lower in tracts classified by the multidimensional disadvantage index compared to the unidimensional disadvantage index and affluence index. For example (Figure 3), at the fourth quintile of disadvantage the predicted prevalence of coronary heart disease was 7.1% (95% CI=6.8, 7.3) when using the multidimensional disadvantage index but was 0.6 percent higher (7.7%; 95% CI=7.5, 7.9) in census tracts classified in the fourth quintile of the reverse affluence index. The pattern was similar for the other conditions (diabetes and obesity), but the confidence intervals overlapped at higher levels of disadvantage. The unidimensional disadvantage index tended to track most closely with the reverse affluence index for the predicted prevalence of diabetes (Figure 2) and coronary heart disease (Figure 3), although the confidence intervals overlapped with both the affluence index and multidimensional disadvantage index. In general, the multidimensional disadvantage index tended to underestimate disease burden in the most disadvantaged census tracts and overestimate disease burden in the least disadvantaged tracts. The unidimensional disadvantage index and the affluence index provided greater precision in identifying the most disadvantaged and least disadvantaged neighborhoods with respect to health status.

## DISCUSSION

Using national data on the neighborhood prevalence of three chronic health conditions in the US, we compared disease prevalence across quintiles of three different disadvantage indices: a multidimensional disadvantage index (including economic, social, and housing variables), a unidimensional disadvantage index (based on indicators of poverty and low income), and a unidimensional index of neighborhood affluence (based on indicators of high income, education, and occupational position). We found that population health at the neighborhood level was patterned differently depending on the index used. In comparison to the multidimensional disadvantage index, the unidimensional affluence and disadvantage indices were better able to differentiate disease prevalence across the gradients in disadvantage.

There are at least 33 publicly available disadvantage indices in the US, which include multiple economic, social, and housing items (up to 29 items in a single index). (13-15) None of these indices distinguish between disadvantage and affluence. Yet, our results provided evidence that disadvantage may be best captured using a more parsimonious set of items with respect to identifying populations at risk for poor health. Compared to the unidimensional disadvantage or affluence indices (each with three variables), the multidimensional disadvantage index (with seven variables) had less precision in distinguish between neighborhoods with highest and lowest disease burden.

Findings have implications for the use of disadvantage indices in public health policy to identify populations most in need of resources. Neighborhood disadvantage indices have recently been used in US health policy to inform vaccine distribution (39) and to account for social risks in Medicare and Medicaid payments (15,40). For example, the Centers for Medicare and Medicaid Services (CMS) Accountable Care Organization Realizing Equity, Access, and Community Health (ACO REACH) program uses the Community Deprivation Index (CDI) to identify disadvantaged areas and provide additional resources to health care providers that serve these areas (41). The CDI is a multidimensional index with 17 variables capturing economic, education, family, household, transportation, and housing domains. However, our findings indicate that using a multidimensional index risks redirecting resources away from communities classified as the least disadvantaged that in fact have higher prevalence of disease in comparison to the least disadvantaged communities as defined by a unidimensional index. For example, in comparison to the unidimensional disadvantage index, using the multidimensional disadvantage index to identify low risk communities would direct resources away from over 4000 census tracts (Table 2a) representing over 16 million people (based on an average tract population of 4000) with higher disease prevalence of coronary heart disease (Supplemental Table 1a). Similarly, using a multidimensional disadvantage index instead of a unidimensional affluence index to identify communities in greatest need of health care would potentially misdirect resources towards over 6000 US census tract neighborhoods (Table 2b) representing over 24 million people, where coronary heart disease prevalence is in fact lower (Supplemental Table 1b). Using a unidimensional disadvantage or affluence index would result in greater precision in identifying communities in greatest need of health care resources.

### Strengths and Limitations

Strengths of this study include the nationwide analysis of all residential census tracts in the United States, and the rigorous evaluation of multidimensional vs. unidimensional indices as they relate to national estimates of neighborhood health conditions in 2022. Despite these strengths, some limitations should be acknowledged. The prevalence of neighborhood health conditions came from the CDC PLACES data, which are model-based estimates using small-area estimation methods (multilevel regression and post stratification) (37). While direct population-based survey estimates would have been ideal for our validity analyses, PLACES provides the only publicly available census tract data on chronic disease prevalence. Moreover, the PLACES model-based estimates have been shown to be strongly correlated with direct survey estimates from both BRFSS data and local survey data at the state, county, and city levels (42-45), supporting the validity of the data. Although the three disadvantage indices used in this study were empirically derived based on a factor analysis, (36) they were operationalized as a mean of all index items. Greater precision might be attained by using factor scores in index construction but could also detract from their ease of interpretation in public health policy and research.

## CONCLUSIONS

The inclusion of multiple social, economic, education, housing, and transportation items in area-based disadvantage indices may be compromising their performance with respect to understanding population health. Our findings suggest that a unidimensional affluence or disadvantage index with a more parsimonious set of items would have greater precision in identifying communities at risk for poor health in the US.

## Figures and Tables

**FIGURE 1.**
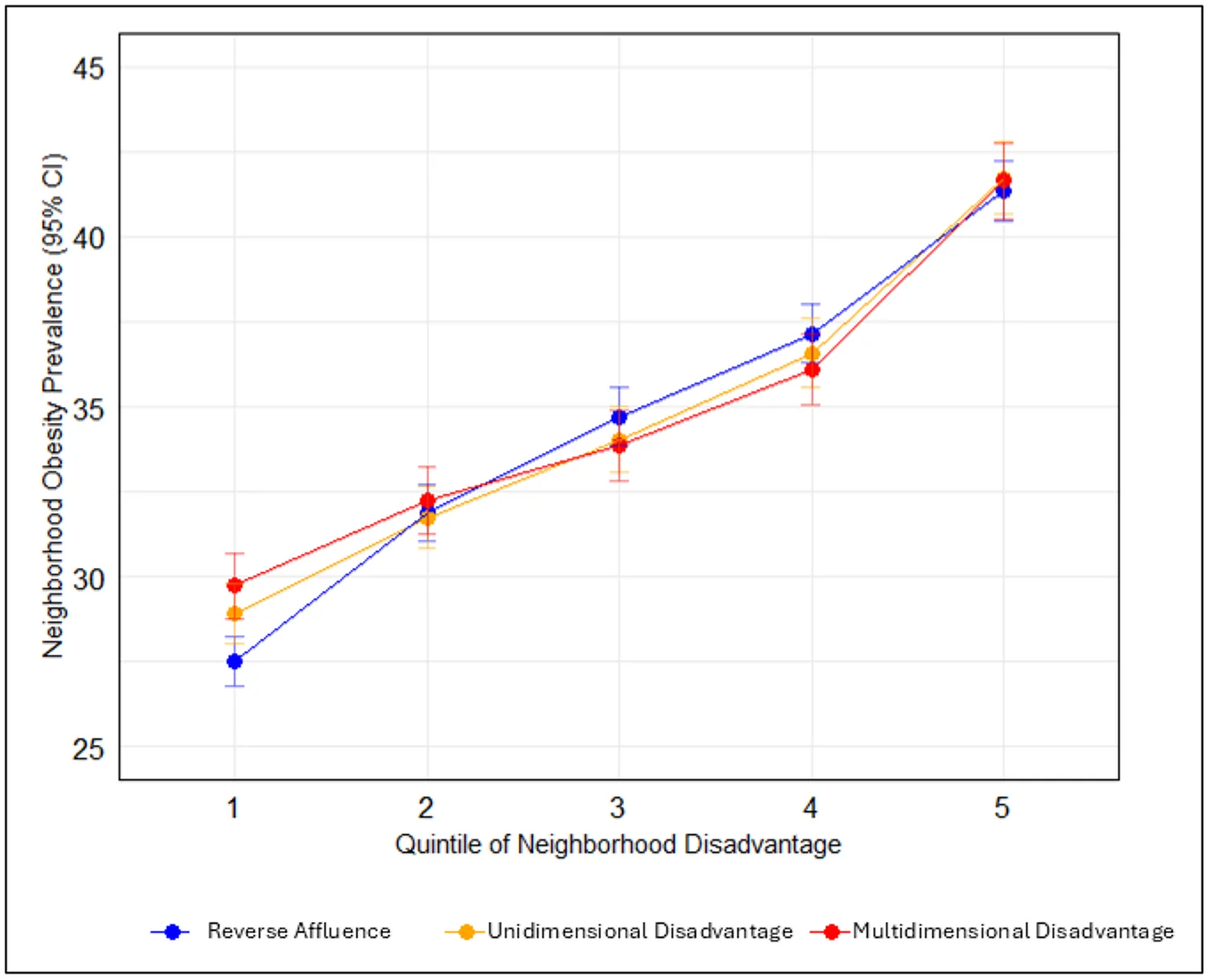
Predicted Census Tract Prevalence (%) of Obesity by Quintiles of Neighborhood Disadvantage Indices (United States, 2022) Note: predicted prevalence is population average effect adjusted for census tract population density. All models include a random effect State Source: CDC PLACES data 2022

**FIGURE 2.**
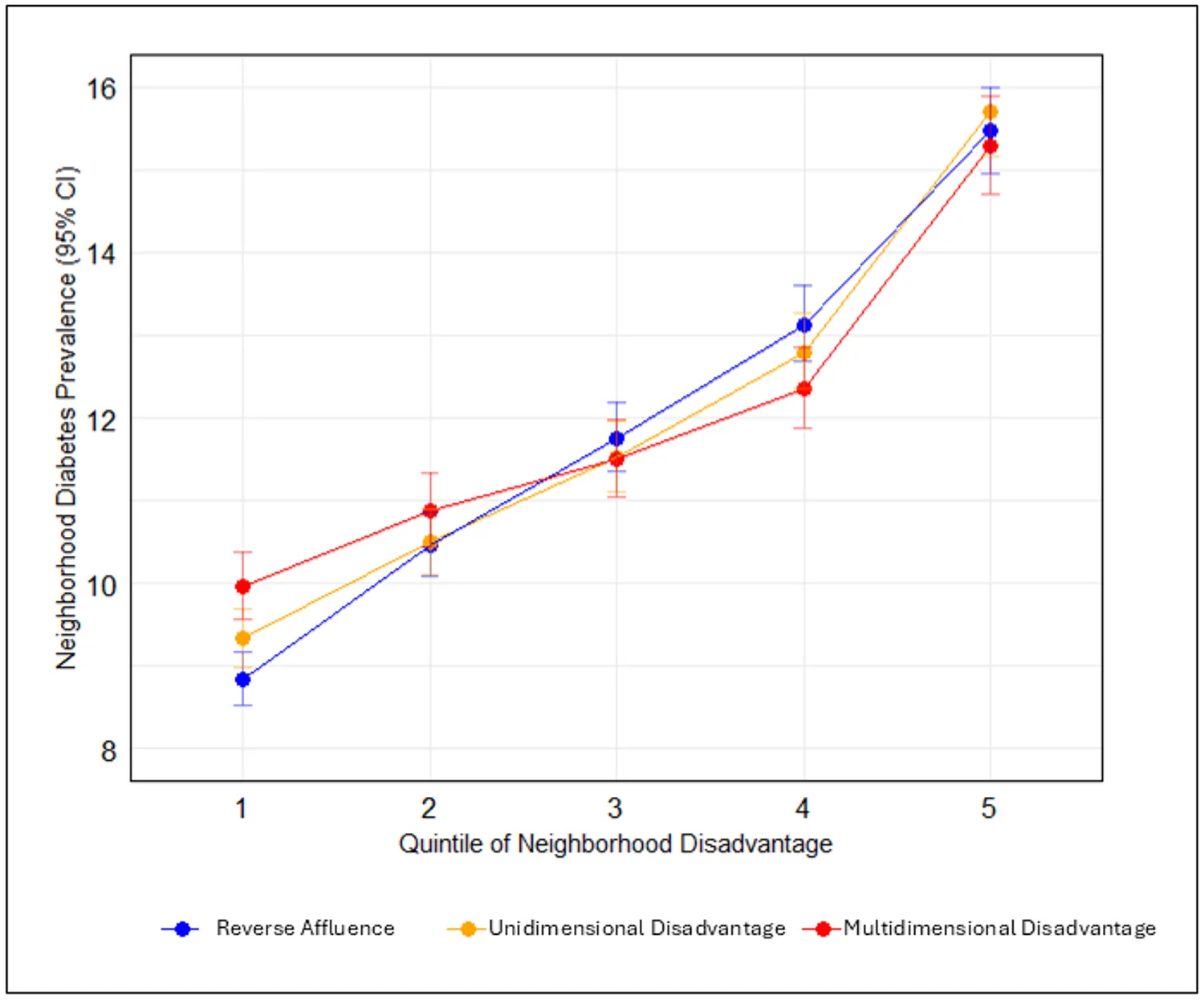
Predicted Census Tract Prevalence (%) of Diabetes by Quintiles of Neighborhood Disadvantage Indices (United States, 2022) Note: predicted prevalence is population average effect adjusted for census tract population density. All models include a random effect State Source: CDC PLACES data 2022

**FIGURE 3.**
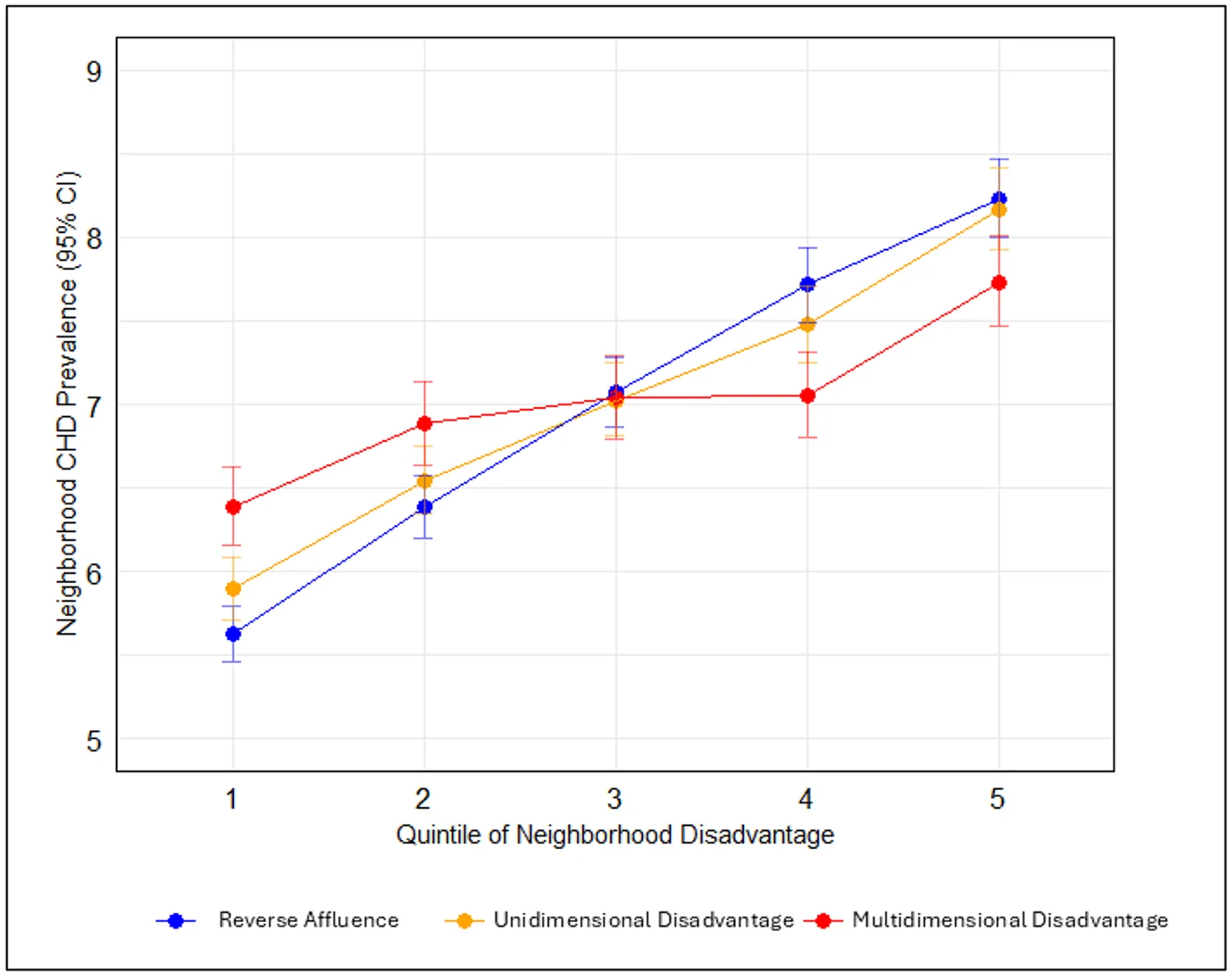
Predicted Census Tract Prevalence (%) of Coronary Heart Disease (CHD) by Quintiles of Neighborhood Disadvantage Indices (United States, 2022) Note: predicted prevalence is population average effect adjusted for census tract population density. All models include a random effect State Source: CDC PLACES data 2022

**Table 1.** Prevalence of Health Conditions by Three Neighborhood Indices: United States 2022 (N=83,522 census tracts)

	Census Tract Prevalence (%) Mean (SD)		
	Obesity	Diabetes	Coronary Heart Disease
Overall	34.4 (7.2)	12.4 (3.8)	7.0 (2.2)
Multidimensional Disadvantage Index			
Q1 (N=16,861)	30.0 (5.2)	10.3 (2.1)	6.5 (1.8)
Q2 (N=16,886)	32.5 (5.7)	11.3 (2.4)	7.0 (2.0)
Q3 (N=16,868)	33.9 (6.2)	12.0 (3.0)	7.1 (2.2)
Q4 (N=16,814)	35.7 (6.6)	12.9 (3.6)	7.1 (2.3)
Q5 (N=16,082)	40.4 (7.4)	15.8 (4.8)	7.5 (2.4)
Unidimensional Disadvantage Index			
Q1 (N=16,821)	28.7 (5.3)	9.6 (2.1)	5.9 (1.7)
Q2 (N=16,856)	31.6 (5.5)	10.8 (2.3)	6.6 (1.9)
Q3 (N=16,848)	34.0 (5.4)	12.0 (2.5)	7.1 (2.0)
Q4 (N=16,839)	36.5 (5.5)	13.4 (2.9)	7.6 (2.2)
Q5 (N=15,972)	41.6 (6.6)	16.5 (4.4)	8.1 (2.3)
Unidimensional Affluence Index (Reversed)			
Q1 (N=16,860)	26.7 (4.7)	9.1 (2.2)	5.4 (1.7)
Q2 (N=16,828)	31.5 (4.7)	10.8 (2.2)	6.4 (1.8)
Q3 (N=16,808)	34.8 (4.8)	12.2 (2.5)	7.1 (1.9)
Q4 (N=16,641)	37.7 (4.9)	13.8 (2.9)	7.9 (2.0)
Q5 (N=16,374)	41.7 (5.8)	16.5 (3.9)	8.3 (2.2)

Source: CDC PLACES data 2022

SD = standard deviation

**Table 2a.** Contingency Table of US Census Tracts by Quintiles of Multidimensional vs. Unidimensional Disadvantage Index: ACS 2018-22, N=84,539

	Unidimensional Disadvantage Index Quintiles						
Multidimensional Disadvantage Index Quintiles	Q1	Q2	Q3	Q4	Q5	Missing	Total
Q1	12435	4192	208	0	0	72	16907
Q2	3254	8167	5181	296	1	9	16908
Q3	967	3656	7525	4681	66	12	16907
Q4	199	842	3738	9256	2840	33	16908
Q5	13	11	216	2635	13961	71	16907
Missing						2	2
Total	16868	16868	16868	16868	16868	199	84539

**Table 2b.** Contingency Table of US Census Tracts by Quintiles of Multidimensional Disadvantage Index and Unidimensional Affluence (Reversed): ACS 2018-22 (N=84,539)

	Unidimensional Affluence Index (Reversed) Quintiles						
Multidimensional Disadvantage Index Quintiles	Q1	Q2	Q3	Q4	Q5	Missing	Total
Q1	8964	5259	2110	482	92		16907
Q2	3753	5092	4798	2822	443		16908
Q3	2477	3441	4500	4717	1772		16907
Q4	1454	2237	3736	5153	4328		16908
Q5	259	878	1764	3733	10272	1	16907
Missing						2	2
Total	16907	16907	16908	16907	16907	3	84539

**Table 2c.** Contingency Table of US Census Tracts by Quintiles of Unidimensional Disadvantage Index and Unidimensional Affluence (Reversed): ACS 2018-22 (N=84,539)

	Unidimensional		Affluence Index (Reversed) Quintiles				
Unidimensional Disadvantage Index Quintiles	Q1	Q2	Q3	Q4	Q5	Missing	Total
Q1	10539	4704	1302	260	63		16868
Q2	4140	6153	4610	1709	256		16868
Q3	1486	3682	5633	4745	1322		16868
Q4	570	1739	3815	6239	4505		16868
Q5	159	611	1538	3944	10615	1	16868
Missing	13	18	10	10	146	2	199
Total	16907	16907	16908	16907	16907	3	84539

## References

[1] Malla G, Long DL, Cherrington A, Goyal P, Guo B, Safford MM, Neighborhood Disadvantage and Risk of Heart Failure: The Reasons for Geographic and Racial Differences in Stroke (REGARDS) Study. Circ: Cardiovascular Quality and Outcomes. 2024 Mar;17(3). doi:10.1161/CIRCOUTCOMES.123.009867 PMC10950536 38328917

[2] Lam TM, Vaartjes I, Grobbee DE, Karssenberg D, Lakerveld J. Associations between the built environment and obesity: an umbrella review. Int J Health Geogr. 2021 Dec;20(1):7. doi:10.1186/s12942-021-00260-6 PMC7852132 33526041

[3] Chandrabose M, Rachele JN, Gunn L, Kavanagh A, Owen N, Turrell G, Built environment and cardio-metabolic health: systematic review and meta-analysis of longitudinal studies. Obesity Reviews. 2019 Jan;20(1):41–54. doi:10.1111/obr.12759 30253075

[4] Diez Roux AV, Mujahid MS, Hirsch JA, Moore K, Moore LV. The Impact of Neighborhoods on CV Risk. Glob Heart. 2016 Sep;11(3):353–63. doi:10.1016/j.gheart.2016.08.002. PMC5098701 27741982

[5] Boardman JD, Finch BK, Ellison CG, Williams DR, Jackson JS. Neighborhood disadvantage, stress, and drug use among adults. J Health Soc Behav. 2001 Jun;42(2):151–65. 11467250

[6] Ross CE, Mirowsky J, Pribesh S. Powerlessness and the amplification of threat: Neighborhood disadvantage, disorder, and mistrust. American Sociological Review. 2001 Aug;66(4):568–91. doi:10.2307/3088923

[7] Cagney KA, Cornwell EY. Neighborhoods and health in later life: The intersection of biology and community. annu rev gerontol geriatr. 2010 Nov 1;30(1):323–48. doi:10.1891/0198-8794.30.323

[8] Diez Roux AV, Mair C. Neighborhoods and health. Annals of the New York Academy of Sciences. 2010 Feb;1186(1):125–45. doi:10.1111/j.1749-6632.2009.05333.x 20201871

[9] Pickett KE, Pearl M. Multilevel analyses of neighborhood socio-economic context and health outcomes: A critical review. Journal of Epidemiology and Community Health. 2001;55:111–22. doi:10.1136/jech.55.2.111 PMC1731829 11154250

[10] Rosso AL, Grubesic TH, Auchincloss AH, Tabb LP, Michael YL. Neighborhood amenities and mobility in older adults. American Journal of Epidemiology. 2013 Sep 1;178(5):761–9. doi:10.1093/aje/kwt032 PMC3817453 23666814

[11] Rollings KA, Dannenberg AL, Frumkin H, Jackson RJ. Built Environment and Public Health: More Than 20 Years of Progress. Am J Public Health. 2024 Jan;114(1):27–33. doi:10.2105/AJPH.2023.307451 PMC10726940 38091569

[12] Lian M, Struthers J, Liu Y. Statistical Assessment of Neighborhood Socioeconomic Deprivation Environment in Spatial Epidemiologic Studies. OJS. 2016;06(03):436–42. doi:10.4236/ojs.2016.63039 PMC4940131 27413589

[13] Trinidad S, Brokamp C, Mor Huertas A, Beck AF, Riley CL, Rasnick E, Use of area-based socioeconomic deprivation indices: A scoping review and qualitative analysis. Health Affairs. 2022;41(12):1804–11. doi:10.1377/hlthaff.2022.00482 36469826

[14] Franchi L, Barreto Parra PN, Chorniy A, Weston BW, Meurer J, Whittle J, A comparative assessment of measures of area-level socio-economic status. SSRN Journal. 2023. doi:10.2139/ssrn.4547382

[15] RAND Health Care. Landscape of Area-Level Deprivation Measures and Other Approaches to Account for Social Risk and Social Determinants of Health in Health Care Payments [Internet]. U.S. Department of Health & Human Services; 2022. Available from: https://aspe.hhs.gov/sites/default/files/documents/8c7f9b317b868fad134dc9001a7e9344/Area-Level-SDOH-Indices-Report.pdf

[16] Lines LM, Long MC, Zangeneh S, DePriest K, Piontak J, Humphrey J, Composite indices of social determinants of health: overview, measurement gaps, and research priorities for health equity. Population Health Management. 2023 Oct;26(5):332–40. doi:10.1089/pop.2023.0106 37824819

[17] Kind AJH, Buckingham WR. Making Neighborhood-Disadvantage Metrics Accessible — The Neighborhood Atlas. N Engl J Med. 2018 Jun 28;378(26):2456–8. doi:10.1056/NEJMp1802313 PMC6051533 29949490

[18] Singh GK. Area Deprivation and Widening Inequalities in US Mortality, 1969–1998. Am J Public Health. 2003 Jul;93(7):1137–43. doi:10.2105/AJPH.93.7.1137 PMC1447923 12835199

[19] Acevedo-Garcia D, Noelke C, McArdle N, Sofer N, Hardy EF, Weiner M, Racial and ethnic inequities in children’s neighborhoods: evidence from the new child opportunity index 2.0. Health Affairs. 2020 Oct;39(10):1693–701. doi:10.1377/hlthaff.2020.00735 33017244

[20] Flanagan BE, Gregory EW, Hallisey EJ, Heitgerd JL, Lewis B. A social vulnerability index for disaster management. Journal of Homeland Security and Emergency Management. 2011 Jan 5;8(1). doi:10.2202/1547-7355.1792

[21] Butler DC, Petterson S, Phillips RL, Bazemore AW. Measures of social deprivation that predict health care access and need within a rational area of primary care service delivery. Health Services Research. 2013;48(2pt1):539–59. doi:10.1111/j.1475-6773.2012.01449.x PMC3626349 22816561

[22] Barber LE, Maliniak ML, Nash R, Moubadder L, Haynes D, Ward KC, A comparison of three area-level indices of neighborhood deprivation and socioeconomic status and their applicability to breast cancer mortality. J Urban Health. 2024 Feb;101(1):75–9. doi:10.1007/s11524-023-00811-1 PMC10897108 38158547

[23] Gladish N, Phillips RL, Rehkopf DH. Estimation of Mortality via the Neighborhood Atlas and Reproducible Area Deprivation Indices. JAMA Netw Open. 2026 Jan 12;9(1):e2546800. doi:10.1001/jamanetworkopen.2025.46800 PMC12797101 41525076

[24] Kaalund K, Thoumi A, Bhavsar NA, Labrador A, Cholera R. Assessment of population-level disadvantage indices to inform equitable health policy. Milbank Q. 2022 Dec;100(4):1028–75. doi:10.1111/1468-0009.12588 PMC9836250 36454129

[25] Limburg A, Rehkopf DH, Gladish N, Phillips RL, Udalova V. Validating 8 Area-Based Measures of Social Risk for Predicting Health and Mortality. JAMA Health Forum. 2025 Aug 15;6(8):e252669. doi:10.1001/jamahealthforum.2025.2669 PMC12357199 40815521

[26] Ramgopal S, Crowe RP, Misra AJ, Cash RE. Comparison of the Social Vulnerability Index, Area Disadvantage Index, and Social Deprivation Index for Adults With Out-of-Hospital Emergencies. Public Health Rep. 2025 Nov;140(5–6):522–30. doi:10.1177/00333549251357818 PMC12373651 40842234

[27] Rollings KA, Noppert GA, Griggs JJ, Ibrahim AM, Clarke PJ. Comparing deprivation vs vulnerability index performance using medicare beneficiary surgical outcomes. JAMA Surg. 2024 Dec 1;159(12):1404. doi:10.1001/jamasurg.2024.4195 PMC11447624 39356528

[28] Lou S, Giorgi S, Liu T, Eichstaedt JC, Curtis B. Measuring disadvantage: A systematic comparison of United States small-area disadvantage indices. Health & Place. 2023 Mar;80:102997. doi:10.1016/j.healthplace.2023.102997 PMC10038931 36867991

[29] Browning CR, Cagney KA. Moving beyond Poverty: Neighborhood Structure, Social Processes, and Health. Journal of Health and Social Behavior. 2003;44(4):552–71. doi:10.2307/1519799 15038149

[30] Wen M, Browning CR, Cagney KA. Poverty, affluence, and income inequality: Neighborhood economic structure and its implications for health. Social Science & Medicine. 2003 Sep;57(5):843–60. doi:10.1016/S0277-9536(02)00457-4 12850110

[31] Massey DS, Brooks-Gunn J, Duncan GJ, Aber L. Back to the future: The rediscovery of neighborhood context. Contemporary Sociology. 1998 Nov;27(6):570. doi:10.2307/2654223

[32] Clarke PJ, Ailshire JA, House JS, Morenoff JD, King K, Melendez R, Cognitive function in the community setting: the neighbourhood as a source of ‘cognitive reserve’? J Epidemiol Community Health. 2012 Aug;66(8):730–6. doi:10.1136/jech.2010.128116 PMC3387518 21515547

[33] Massey DS. The age of extremes: Concentrated affluence and poverty in the twenty-first century. Demography. 1996 Nov 1;33(4):395–412. doi:10.2307/2061773 8939412

[34] Christine PJ, Moore K, Crawford ND, Barrientos-Gutierrez T, Sánchez BN, Seeman T, Exposure to neighborhood foreclosures and changes in cardiometabolic health: Results from MESA. Am J Epidemiol. 2017 Jan 15;185(2):106–14. doi:10.1093/aje/kww186 PMC5253973 27986705

[35] Clarke P, Melendez R, Noppert G, Chenoweth M, Gypin L. National Neighborhood Data Archive (NaNDA): Socioeconomic Status and Demographic Characteristics of Census Tracts and ZIP Code Tabulation Areas, United States, 1990-2022: Version 5 [Internet]. ICPSR - Interuniversity Consortium for Political and Social Research; 2022 [cited 2025 May 5]. Available from: https://www.icpsr.umich.edu/web/ICPSR/studies/38528/versions/V5 doi:10.3886/ICPSR38528.V5

[36] Clarke P, Melendez R. The national neighborhood data archive (NaNDA): 2016-2020 neighborhood sociodemographic measures technical documentation on index creation [Internet]. 2023 Apr 18. doi:10.7302/7033

[37] Greenlund KJ, Lu H, Wang Y, Matthews KA, LeClercq JM, Lee B, PLACES: Local data for better health. Prev Chronic Dis. 2022 Jun 16;19:210459. doi:10.5888/pcd19.210459 PMC9258452 35709356

[38] Centers for Disease Control and Prevention. PLACES: Local Data for Better Health, County Data 2024 release [Internet]. 500 Cities & Places; 2024 [cited 2025 May 5]. Available from: https://data.cdc.gov/500-Cities-Places/PLACES-Local-Data-for-Better-Health-County-Data-20/swc5-untb/about_data

[39] Srivastava T, Schmidt H, Sadecki E, Kornides ML. Disadvantage Indices Deployed to Promote Equitable Allocation of COVID-19 Vaccines in the US: A Scoping Review of Differences and Similarities in Design. JAMA Health Forum. 2022 Jan 21;3(1):e214501. doi:10.1001/jamahealthforum.2021.4501 PMC8903102 35977227

[40] Phillips RL, Ostrovsky A, Gilfillan R, Price D, Bazemore AW. Accounting for Social Risks in Medicare and Medicaid Payments [Internet]. 2023. doi:10.26099/DMHF-E064

[41] Robst J, Cogburn R, Forlines G, Frazier L, Kautter J. The development of the Community Deprivation Index and its application to accountable care organizations. Health Affairs Scholar. 2024 Dec 1;2(12):qxae161. doi:10.1093/haschl/qxae161 PMC11629994 39664484

[42] Wang Y, Holt JB, Zhang X, Lu H, Shah SN, Dooley DP, Comparison of methods for estimating prevalence of chronic diseases and health behaviors for small geographic areas: Boston validation study, 2013. Prev Chronic Dis. 2017 Oct 19;14:170281. doi:10.5888/pcd14.170281 PMC5652237 29049020

[43] Wang Y, Holt JB, Xu F, Zhang X, Dooley DP, Lu H, Using 3 health surveys to compare multilevel models for small area estimation for chronic diseases and health behaviors. Prev Chronic Dis. 2018 Nov 1;15:180313. doi:10.5888/pcd15.180313 PMC6219847 30388068

[44] Zhang X, Holt JB, Lu H, Wheaton AG, Ford ES, Greenlund KJ, Multilevel regression and poststratification for small-area estimation of population health outcomes: A case study of chronic obstructive pulmonary disease prevalence using the behavioral risk factor surveillance system. American Journal of Epidemiology. 2014 Apr 15;179(8):1025–33. doi:10.1093/aje/kwu018 24598867

[45] Zhang X, Holt JB, Yun S, Lu H, Greenlund KJ, Croft JB. Validation of multilevel regression and poststratification methodology for small area estimation of health indicators from the behavioral risk factor surveillance system. Am J Epidemiol. 2015 Jul 15;182(2):127–37. doi:10.1093/aje/kwv002 PMC4554328 25957312

